# Giant mixed Sertoli-Leydig-Granulosa sex cord tumor of the testis; clinical, histopathological, and radiological features: a case report

**DOI:** 10.11604/pamj.2017.27.51.10571

**Published:** 2017-05-19

**Authors:** Sakher Tahaineh, Rawan Abu Mughli, Moayid Fallatah

**Affiliations:** 1 Urology Department, Security Forces Hospital, Makkah, Saudi Arabia; 2 Radiology Department, Security Forces Hospital, Makkah, Saudi Arabia; 3 College of Medicine, Umm Al-Qura University, Makkah, Saudi Arabia

**Keywords:** Orchiectomy, sex cord-Gonadal stromal tumors, testicular neoplasms

## Abstract

Sex cord tumors of the testis in post pubertal men are rare. Mixed leydig-Sertoli-Granulosa sex cord tumors are exceptionally rare. To the best of our knowledge there are only three reported similar cases in the literature. We reported a case of a 27-year-old male who presented with huge left scrotal mass of 6-years duration. The gross tumor specimen after resection measured 11 cm in diameter. Histological examination revealed mixed sex cord stromal tumor. This case demonstrates the limited ability of accurate diagnostic determination preoperatively, with pathologic examination and immune-histochemical staining post-orchiectomy representing the only definitive means of diagnosis. It also highlights the unique radiological appearances of this tumor, which were not previously reported in literature.

## Introduction

Sex cord tumors of the testis in post pubertal men are rare [1,2]. Mixed leydig-Sertoli-Granulosa sex cord tumors, are exceptionally rare, with only three reported cases in literature [1,3,4]. We report a case of a 27-year-old male with a huge mixed sex cord tumor of testis, which has a unique presentation of a long standing left scrotal mass of 6-years duration. An outline of this case clinical, radiological and pathological features are presented.

## Patient and observation

**Figure 1:**
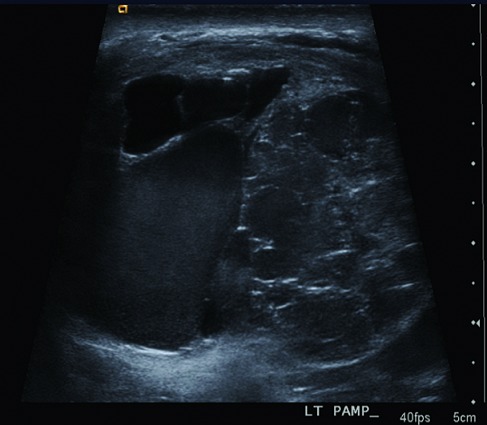
Scrotal ultrasound shows mixed solid and multilocular cystic mass, containing cysts of varying echogenicties

**Figure 2:**
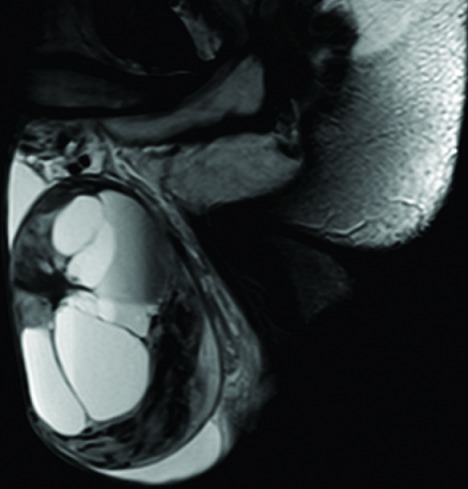
Sagittal T2- weighted MRI of the scrotum shows large intratesticular multilocular complex cystic lesion. Some of the cysts showed fluid fluid levels. The remaining testicular tissue appears of heterogeneous hyperintense signal with mild surrounding hydrocele

**Figure 3:**
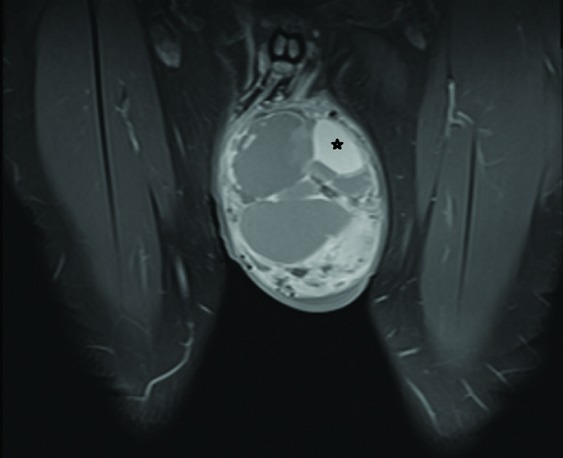
Coronal T1 post contrast images shows heterogenous enhancement of the remaining peripherally displaces testicular tissue. And a cyst that showed hyper intense signal on both T1 pre contrast and T2, in keeping with a hemorrhagic cyst(star) at the superolateral aspect of the mass

**Figure 4:**
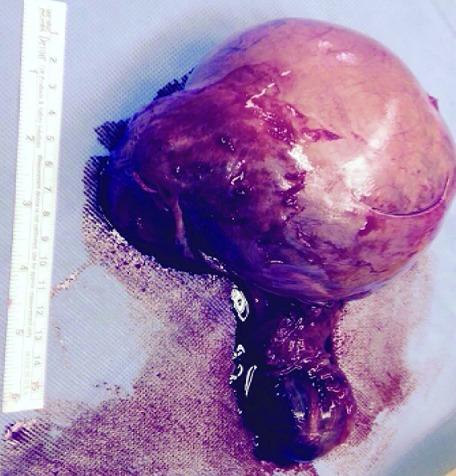
Gross specimen of the testicular tumor after removal from the patient

A 27-year-old man presented to our urology clinic with left testicular swelling of 6-years duration. His medical history was negative, particularly for cryptorchidism. On physical examination, there was a large non tender swelling of the left scrotum, which showed partial transillumination. There was no gynecomastia or any systemic symptoms. Accordingly, ultrasound testicular examination was done; this showed large mixed solid and multilocular cystic mass replacing the left testis. The cysts vary in appearance from anechoic to echogenic (Figure 1). Moreover, grade three left varicocele was noted. Laboratory studies including tumor markers- lactate dehydrogenase, beta human chorionic gonadotropin, estrogen, progesterone and alpha-fetoprotein levels- as well as complete blood count and kidney function tests were all within normal ranges. MRI was recommended for further characterization of the mass. This showed large complex cystic lesion replacing large areas of the central left testis measuring 11* 8.5 cm. The lesion showed hypointense signal on T1 weighted images and hyperintense signal on T2 weighted images (Figure 2) with a part of high signal on both T1 and T2 images at its superolateral aspect in keeping with a hemorrhagic component. Post contrast images also showed heterogeneous enhancement of the remaining testicular tissue at its peripheral inferior part (Figure 3). Next day, additional staging CT scan showed no evidence of extra-testicular metastasis, and the patient was consented for left radical orchiectomy. Due to large size of the mass, simple trans-inguinal orchiectomy was not possible. Instead, he underwent trans-inguinal and trans-scrotal orchiectomy (Figure 4). Histopatholgic examination showed sex cord stromal tumor of mixed forms composed of three cell types; leydig, sertoli, and granulosa cells. Furthermore, tumor margins, spermatic cord, tunica, and scrotal skin were free. Immunohistochemistry was positive for Vimetin, calretinin, and inhibin. Due to negative tumor margins and absence of extra-testicular metastasis, no further management was advised. At 24 months follow up; the patient was doing well; an abdomen and pelvis CT scan was normal.

## Discussion

Testicular cancer is relatively rare, accounting about 1-1.5% of all cancers in men [2,5] however, it is the commonest malignancy in the 15-34 years-old age group [2]. The majority of testicular cancers are germ cell tumors [2,5], while sex cord stromal tumors are rare, representing 4%-6% of all testicular neoplasms [1,6]. The prevalence of sex cord tumors tends to be higher in pediatric patients with a frequency of 10-30% [2]. Sex cord stromal tumors include; leydig, sertoli, granulosa, and theca cell tumors. Also included are tumors that have mixed cell types (which may present as a mixture of any combination of the mentioned cell types) and undifferentiated tumors [6]. Amongst these, leydig cell tumor is the most common histologic type [1,6]. The remaining histologic types are much less common [6]. Mixed sex cord tumor containing all three cell elements; granulosa-sertoli-leydig cell testicular tumor is very rare with only three published cases in literature [1,3,4]. The commonest presentation of testicular tumors is a painless scrotal mass. In some cases, testicular neoplasm may initially be misdiagnosed as orchitis [2]. Although 90% of non germ cell testicular tumors are benign, unfortunately, no radiologic criteria allow differentiation of benign from malignant disease. Consequently, orchiectomy is performed in all cases [2]. Ultrasonography is the primary imaging modality for investigating testicular lesions. The primary goal is the mass localization (intratesticular or extratesticular), and further lesion characterization (cystic or solid) [2]. Magnetic resonance imaging (MRI) may provide additional information in cases where the Ultrasonography results are inconclusive [2,7] and often affecting patient management [7]. To our knowledge, no descriptions of the MRI appearances of a mixed sertoli-ledyig-granulosa cell tumor have been reported. On the other hand, radiological appearances of leydig sex cord tumor and sertoli sex cord tumors were previously described [2,7]. In our case the imaging findings were those of a complex multilocular cystic mass with necrotic and hemorrhagic components. However, larger study sample is needed to characterize the radiological findings of mixed sex cord stromal tumors. When it comes to tumor markers, Inhibin alpha is the most useful immunohistochemical stain for identifying tumours of the sex cord stromal category. Expression of calretinin and vimentin are also common which is concordant with our results [8]. The optimal treatment of sex cord tumors is debatable. This is due to the infrequency of these tumors, and the low percentage of malignant lesions [6]. The standard treatment method is inguinal orchiectomy. Testis sparing surgery can be considered in certain conditions. There is no evidence to support additional therapy in patients with disease clinically confined to testicle. Follow up should be individualized according to patient status and the tumor aggressiveness properties. Given the lack of extra testicular invasion, and the negative metastatic work up, it was reasonable to treat this patient with orchiectomy alone. The patient was followed for two years with out recurrence of the disease.

## Conclusion

Although mixed sex cord tumors of the testis are rare in post pubertal men, they should be considered in the differential diagnosis of testicular masses in adults. The diagnosis can be challenging, as it can present with a long history of testicular mass reaching large sizes. This case reflects the clinical and radiological features of mixed sex cord tumor. It further illustrates the difficulty of differentiating it clinically and radiologically from other lesions. Also it highlights the tumor potential for assuming extreme proportions. Given the rarity of these tumors, and its challenging imaging features, more data collection will be helpful in the management of future patients.

## Competing interests

The authors declare no competing interest.
